# Discovery of the Micrococcus of Syphilis

**Published:** 1881-09

**Authors:** 


### Discovery of the Micrococcus of Syphilis.

Dr. Aufrecht, of Magdeburg, (*Centralblatt fuer die Med. Wiss.*, No. 13, 1881), announces that he has discovered in syphilitic condylomota a micrococcus, which may be recognized by the following characters. The single cocci are of rather coarse grain; they are generally of the form of diplococci, or two joined together, and the number of these is greater than of the single cocci. They are very seldom in threes. They are stained deeply by fuchsin. He has found them in six cases; but in one, where the condyloma was ulcerated, and, in another, where it had been painted with corrosive sublimate, they were very scarce. He, therefore, excludes ulcerated condylomata, or those which have been treated specifically. To obtain the micrococci, the condyloma should be incised with a lancet, and the blood sponged away; then a drop of the serous fluid that follows should be collected on a cover-glass, which is put under a bell-jar for twenty-four hours, to dry. At the end of that time, a drop of a half per mille solution of fuchsin is placed on an object-glass, and the cover-glass is laid on it. The excess of fuchsin is wiped away after two or three minutes, and the object examined with Hartnack's 9A immersion-lens. To preserve the object, he puts a little damar varnish around the edge of the cover-glass.—*London Med. Record, June 15, 1881.*

